# Salaries, degrees, and babies: Trends in fertility by income and education among Japanese men and women born 1943–1975—Analysis of national surveys

**DOI:** 10.1371/journal.pone.0266835

**Published:** 2022-04-27

**Authors:** Cyrus Ghaznavi, Haruka Sakamoto, Lisa Yamasaki, Shuhei Nomura, Daisuke Yoneoka, Kenji Shibuya, Peter Ueda

**Affiliations:** 1 Department of Health Policy and Management, School of Medicine, Keio University, Tokyo, Japan; 2 Medical Education Program, Washington University School of Medicine, St. Louis, Missouri, United States of America; 3 Department of Global Health Policy, Graduate School of Medicine, The University of Tokyo, Tokyo, Japan; 4 Tokyo Foundation for Policy Research, Tokyo, Japan; 5 School of Medicine, Nagasaki University, Nagasaki, Japan; 6 Infectious Disease Surveillance Center at the National Institute of Infectious Diseases, Tokyo, Japan; 7 Clinical Epidemiology Division, Department of Medicine, Solna, Karolinska Institutet, Stockholm, Sweden (PU); Universiti Malaysia Sabah, MALAYSIA

## Abstract

**Background:**

While fertility rates have decreased during the second half of the 20^th^ century in Japan, little is known regarding trends in the number of children that men and women have across birth cohorts and whether these differ by education and income.

**Methods:**

We used data from four rounds of the National Fertility Survey (1992, 2005, 2010 and 2015) and included men and women aged 40–49 years (16728 men and 17628 women). By 5-year birth cohorts, we assessed the distribution of number of children (0, 1, 2 and 3 or more) and total fertility (the mean number of children) at completed fertility (age 45–49 or 40–44 years depending on birth cohort). We assessed trends in these fertility outcomes in men and women separately, and by education (no university education; university education) for men and women and by reported annual income (0 to <3 000 000 JPY; 3 000 000 to <6 000 000 JPY; ≥6 000 000 JPY) for men.

**Results:**

When comparing those born in 1943–1948 with those born in 1971–1975, the proportion with no children had increased from 14.3 to 39.9% for men and from 11.6 to 27.6% for women. This increase coincided with a decrease in the proportions of individuals with 2 or more children. Total fertility had decreased from 1.92 to 1.17 among men and from 1.96 to 1.42 among women. For men, those with a university degree were more likely to have children than those without a university degree in all birth cohorts except 1943–1947. Men with higher income were more likely to have children across birth cohorts. While the proportion who had children had decreased in all income groups, the decrease was steeper among those in the lowest income group. Among women born 1956–1970, those with a university degree were less likely to have children than those without a university degree; this difference was no longer seen among those born 1971–1975. For both men and women, trends in having children and total fertility across birth cohorts did not differ by educational status.

**Conclusions:**

The decline in the total fertility rate in Japan can be attributed to both an increasing proportion of the population who have no children and a lower number of children among those who have children. Men with lower education and income were less likely to have children and the disparity in the number of children that men have by income had increased in more recent birth cohorts. Among women, higher education was associated with lower fertility, although this pattern was no longer observed among those born in 1971–1975.

## Introduction

In 2020, around 840000 Japanese children were born in Japan, which is the lowest number recorded since the collection of national fertility data began in 1899. In the same year, the total fertility rate was 1.36, and the Japanese population is predicted to decrease by one-third by 2060 [1]. While the low birth rates are a central challenge facing the country and numerous policies have been implemented with the aim to increase fertility [2], important knowledge gaps remain regarding historical and contemporary fertility outcomes in the Japanese population.

It is widely known that the total fertility rate, defined as the mean number of children born per woman in reproductive age, has decreased in the second half of the 20^th^ century after which it has stabilized at a low level. However, it is unclear whether the decrease was attributable to a larger proportion of the population being childless, a lower number of children being born to those who have children, or both. Moreover, conflicting hypotheses have been suggested regarding a potential association of socioeconomic status, including education and income, with fertility. On the one hand, lower income may be associated with lower fertility. This is because the likelihood of being married or being in a romantic relationship is strongly correlated with higher income among Japanese men [3–5] and the cost of raising children may prevent financially strained couples from having the number of children they want [6–8]. On the other hand, difficulties in combining full-time work with child-rearing is thought to deter some women, especially those with high education, from having children [6, 7]. Despite being subject to much speculation, it remains unknown whether the number of children differ across levels of education and income and how this may have changed across birth cohorts.

In this study, we used data from four rounds of the National Fertility Survey to describe how the distribution of the number of children and total fertility have changed among individuals born 1943–1975 and whether such trends differ by level of education for men and women, and income for men. The analysis of income was not performed for women, as the level of income was reported at the time of survey participation and many Japanese women work shorter hours or become homemakers after marriage and childbirth [9]. In addition, we assessed the association of an expanded set of sociodemographic variables with fertility outcomes in the youngest birth cohort (1971–1975).

## Methods

### Data sources

The National Fertility Survey of Japan (described in detail elsewhere [10, 11]) is carried out by The National Institute of Population and Social Security Research (IPSS), under the Japanese Ministry of Health, Labour and Welfare, to collect nationally representative data on subjects related to marriage and childbirth. In each survey, cluster sampling was employed with districts in the Population Census of Japan as primary sampling units to conduct two national sub-surveys: one for married couples in which the wife was aged 16 to <50 years, with the wife providing information about the husband, and another for unmarried individuals aged 18–49. A self-administered questionnaire was given to participants during a home visit; upon completion, the questionnaire was returned in a sealed envelope during a follow-up visit. In the surveys from 1992, 2005, 2010 and 2015, which were used in this study, the valid response rate ranged between 70.0 and 77.7% (mean 74.6%) among unmarried respondents and 85.7 and 91.1% (mean 87.8%) among married couples.

Data regarding the number of individuals in the Japanese population by age, sex, and marital status was obtained from the Population Census of Japan (1990–2015) [12]. We used this information for the calculation of sample weights to account for non-response, as described below.

### Fertility outcomes

We assessed two outcomes aimed at capturing information on completed fertility in each birth cohort: (1) the distribution of the number of children (with a focus on those with no children, commonly referred to as *childlessness* [13–15]) and (2) total fertility (mean total number of children per individual) [13, 16–20]. A key consideration regarding these fertility outcomes is: at which age fertility should be regarded as completed, as some women and men have children later in their life stage. The older the age at which fertility outcomes are assessed, the better completed fertility is captured, particularly for men. If fertility is assessed at an older age, however, the more recent birth cohorts would not be included in the analyses. As such, most previous studies have used fertility outcomes at ages between 40 and 45 in their definitions of cohort total fertility and childlessness [16, 19, 20]. As we used data from national surveys conducted approximately every 5 years, we needed to include a broader age range in order to capture the completed fertility for all birth years in the investigated birth cohorts. Against this background, we decided to use data on fertility outcomes at age 45–49 years and supplemented this with fertility outcome data from age 40–44 years.

### Study population

We used the National Fertility Survey of 1992, 2005, 2010 and 2015. The surveys from 1997 and 2002 were not used because they did not include information regarding the number of children among unmarried individuals. We assessed fertility outcomes in 5-year birth cohorts corresponding to the birth years of the participants who were aged 40–44 years or 45–49 years in each survey round (S1 File [S1 Table]): 1943–1947, 1948–1952, 1956–1960, 1961–1965, 1966–1970 and 1971–1974. Individuals born in 1953–1955 were not included as their data in the available surveys did not correspond to the assessed age range (S1 File [S1 Table]). Both unmarried and married participants were asked regarding the number of children in their current or previous relationships, as described in the S1 File. We excluded participants with unknown number of children. The proportion of excluded participants ranged from 1.7% to 2.6% for women and from 1.3% to 6.7% for men (S2 Table in S1 File). The final study population included 16 728 men (13 786 married) and 17 628 women (14 896 married); the number of participants by birth cohort and survey are shown in S1 File (S3 Table). Sample weights were used to adjust for differential probabilities of non-response, by age, sex, and marital status and were calculated based on data from the Population Census of Japan, as described in detail elsewhere [5, 21] and in the S1 File.

### Statistical methods

All analyses were performed separately for men and women using Stata version 15.0 (StataCorp LP, College Town, TX). P-values of < 0.05, odds ratio (OR) 95% confidence intervals that did not include 1 and 95% confidence intervals of coefficients in linear regressions that did not include 0 were considered as statistically significant. The Regional Ethics Committee at The University of Tokyo, Japan approved the study.

#### Trends in number of children and total fertility across birth cohorts

First, we assessed trends in fertility outcomes across birth cohorts. In each birth cohort separately, we estimated the distribution of the number of children (0, 1, 2 and 3 or more) and total fertility. In the primary analyses, we used fertility at age 45–49 years in birth cohorts 1943–1947, 1956–1960, 1961–1965, and 1966–1970 and at age 40–44 years in birth cohorts 1948–1952 and 1971–1975. We assessed trends in each category of number of children using logistic regression with the investigated category of number of children as a binary dependent variable (e.g. when assessing trends in the proportion with 0 children, we used an outcome variable for which the value was set to 1 for those with 0 children and to 0 for the remaining individuals) and birth cohort as a continuous independent variable (accounting for the irregular time interval between the 5-year birth cohorts as described in the S1 File). We assessed trends in total fertility across birth cohorts by using linear regression with total fertility as a continuous dependent variable and birth cohort as a continuous independent variable. To assess whether the results of the primary analysis would be consistent with those of analyses restricted to fertility outcomes assessed at age 40–44 or 45–49 years, we also performed two separate analyses for fertility outcomes registered at each of the two age ranges.

#### Trends in childlessness and total fertility across birth cohorts by educational status and income

Next, we assessed trends in fertility outcomes by educational level for men and women and by annual income for men. We chose to conduct analyses by subgroups based on these variables because they constitute key socioeconomic indicators and because they have been correlated with fertility [13, 14, 16–20, 22] and related outcomes [5, 21, 23] in previous studies. As level of income was reported at the time of survey participation and many Japanese women work shorter hours or become homemakers after marriage and childbirth [9], we did not assess this variable for women. Educational status was categorized into university degree and no university degree. Annual income was categorized into 0 to <3000000 JPY; 3000000 to <6000000 JPY; and ≥6000000 JPY. In each birth cohort separately, we described the proportion of men and women by educational status and the proportion of men by income group. In each birth cohort, we then described the distribution of the number of children and total fertility by education and income group and assessed differences in the distribution of children using *F* statistics for two-way tables and total fertility using the Student’s t-test. In each subgroup by education and income separately, we examined trends in the proportion with no children (childlessness) using logistic regression with having no children as a binary dependent variable and birth cohort as a continuous independent variable and trends in total fertility using linear regression with total fertility as a continuous dependent variable and birth cohort as a continuous independent variable. We assessed whether the trends differed between subgroups by education and income using an interaction term between birth cohort and subgroup category.

#### Association of sociodemographic variables with fertility outcomes in the youngest birth cohort (1971–1975)

Next, we focused on the youngest birth cohorts for which data were available: those born 1971–1975. By the number of children, we presented detailed population characteristics using variables including education, occupational status, annual income, hours worked/week, region of residence, and population size/density of residence; the definitions and categorizations of these variables are shown in S1 File (S4 Table). We then used logistic regression to estimate age-adjusted odds ratios (aORs) for the association of the selected variables (independent variables) with childlessness and having 3 or more children, respectively.

## Results

### Trends in number of children and total fertility across birth cohorts

**Fig 1:**
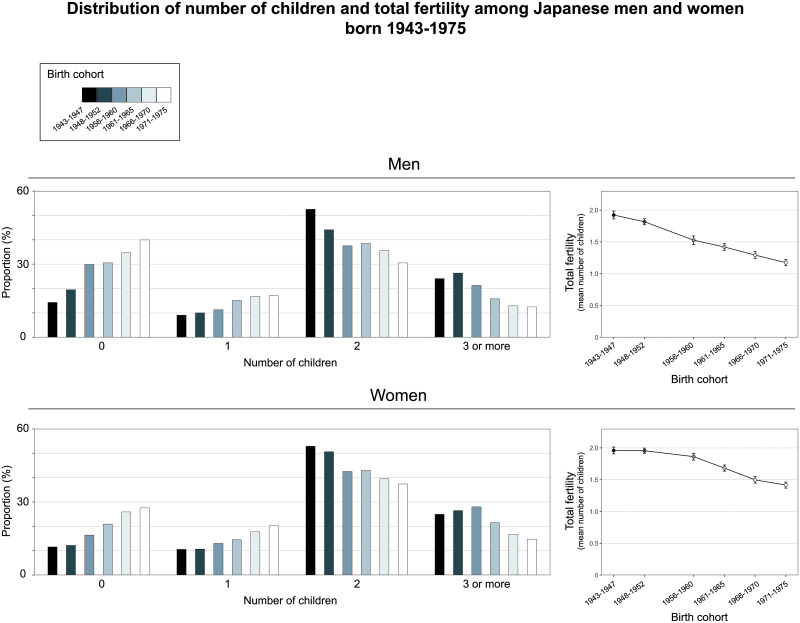
Trends in the distribution of number of children and total fertility across birth cohorts.

S5 and S6 Tables in the S1 File show the distribution of children and total fertility (mean number of children) across birth cohorts. Fig 1 shows trends in fertility outcomes across birth cohorts in the primary analyses. The analyses of trends in the distribution of the number of children showed that the proportion who were childless had increased from 14.3% among those born 1943–1947 to 39.9% among those born 1971–1975 for men (OR for change per 5-year birth cohorts 1.26 (95% CI 1.23 to 1.30)) and from 11.6% among those born 1943–1947 to 27.6% among those born 1971–1975 for women (OR for trend 1.23 (95% CI 1.20 to 1.27)). Moreover, the proportion of men and women with 1 child had increased while the proportion with 2 and 3 or more children had decreased (Fig 1 and S1 File [S5 Table]). Total fertility had decreased, from 1.92 (95% CI 1.86 to 1.98) among those born in 1943–1947 to 1.17 (95% CI 1.13 to 1.22) among those born in 1971–1975 for men (change per 5-year birth cohort: -0.14 (95% CI -0.15 to -0.13)) and from 1.96 (95% CI 1.91 to 2.01) among those born in 1943–1947 to 1.42 (95% CI 1.37 to 1.46) among those born in 1971–1975 for women (change per 5-year birth cohort: -0.10 (95% CI -0.12 to -0.09). Trends in fertility outcomes were similar to those observed in the primary analyses when restricting the analyses to fertility outcomes assessed at age 40–44 years and 45–49 years, respectively (S1 File [S5 and S6 Tables]).

### Trends in childlessness and total fertility across birth cohorts by educational status and income

**Fig 2:**
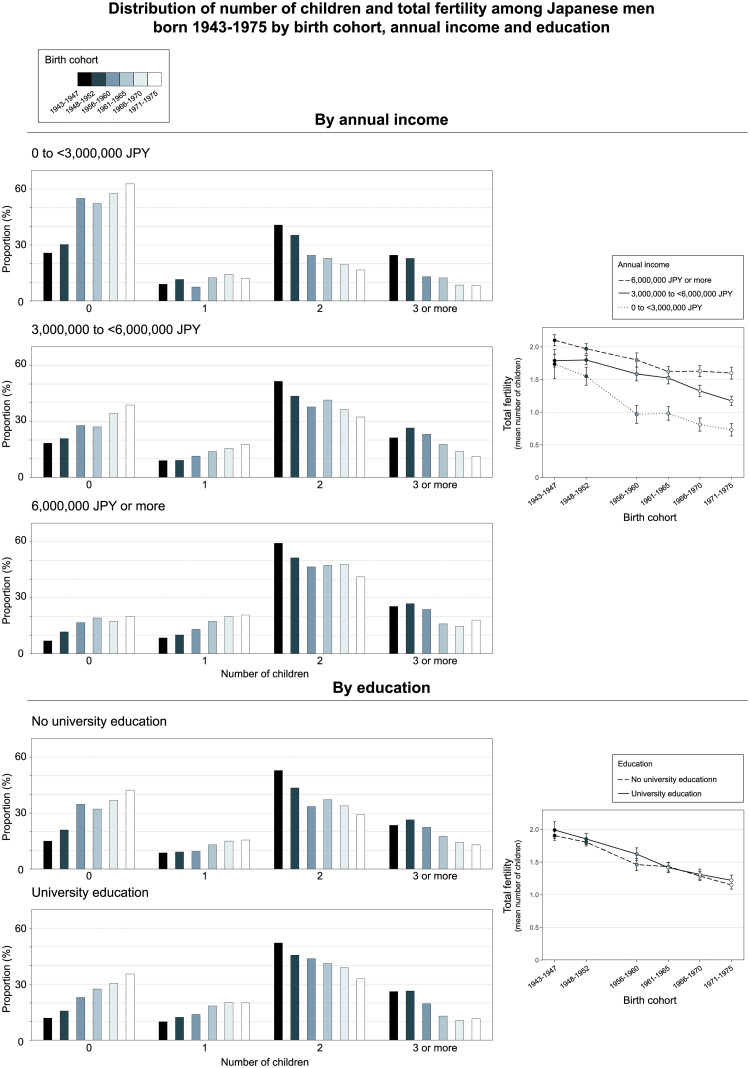
Trends in the distribution of number of children and total fertility across birth cohorts by income group and education in men.

**Fig 3:**
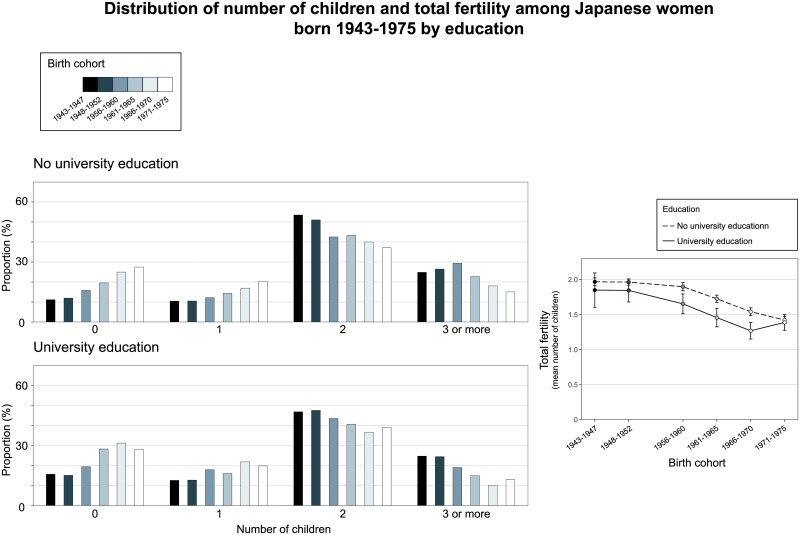
Trends in the distribution of number of children and total fertility across birth cohorts by education in women.

S1 Fig in the S1 File shows the proportion of men and women by educational status and the proportion of men by annual income in each birth cohort. Fig 2 shows the distribution of number of children and total fertility across birth cohorts by educational status and income group for men and Fig 3 shows these fertility outcomes by educational status for women (raw data in S1 File [S7-S9 Tables]). For men, those with a university degree were more likely to have children across birth cohorts except the oldest one (1943–1947), although differences in total fertility were only statistically significant among those born in 1956–1960. In the birth cohorts 1956–1960, 1961–1965 and 1966–1970, women with a university degree were less likely to have children and had lower total fertility as compared with those without such a degree. Among women born in 1943–1947 and 1948–1952 and in the latest birth cohort (1971–1975), there were no statistically significant differences for these fertility outcomes between those with and without university education.

For both men and women, the proportion who did not have any children had increased, and total fertility had decreased in both education groups, with these trends being similar in both groups (Figs 2 and 3 and S1 File [S9 Table]).

In the analyses by income group, men with lower income were less likely to have children as compared with the higher income groups, with this pattern being consistent across birth cohorts. Similarly, total fertility was consistently higher for the highest income group vs the lowest. When comparing those who were born in 1943–1947 vs 1971–1975 the proportion childless had increased from 25.7 to 62.8% and total fertility had decreased from 1.74 to 0.73 in the lowest income group. The corresponding numbers for the highest income group was 6.9% vs 20.0% and 2.10 vs 1.60. Accordingly, childlessness had increased more steeply among those in the lowest income group (OR for trend across birth cohorts 1.32 [9,5% CI 1.23 to 1.43]) as compared with the highest income group (OR 1.21 [95% CI 1.14 to 1.29], p-value for interaction term vs lowest income group 0.044). Similarly, the decrease in total fertility was more pronounced in the lowest income group as compared to the other income groups.

### Association of sociodemographic variables with fertility outcomes in the youngest birth cohort (1971–1975)

The characteristics of men and women born in 1971–1975 by their number of children are shown in S1 File (S10 Table), and aORs for the association of these characteristics with the likelihood of having no children and having 3 or more children, respectively, are shown in Tables 1 and 2. Among men, the likelihood of having children and having 3 or more children increased in a stepwise fashion with increasing income. Men who were unemployed and men who had part-time or temporary employment were less likely to have children and to have 3 or more children, as compared to men with regular employment. Among women, those who had regular employment were the least likely to have children and to have 3 or more children. Women living in cities with over 1000 000 inhabitants were less likely to have children and to have 3 or more children as compared with women living in non-densely inhabited districts. Men living in cities with over 1000 000 inhabitants were less likely to have 3 or more children as compared with men living in non-densely inhabited districts.

**Table 1:** Association of sociodemographic factors with childlessness (having no children) and having 3 or more children among men born 1971–1975.

	Men (n = 2232)			
	No children		3 or more children	
	Row %	aOR (95% CI)	Row %	aOR (95% CI)
*Education*				
No university degree	42.2	Ref.	13.0	Ref.
University degree	35.5	0.75 (0.61 to 0.92)	11.6	0.88 (0.66 to 1.17)
*Occupation*				
Regular employee	34.0	Ref.	13.2	Ref.
Part-time or temporary worker	76.4	6.31 (4.06 to 9.80)	3.2	0.22 (0.07 to 0.64)
Business owner or member of family business	32.8	0.95 (0.67 to 1.33)	16.8	1.33 (0.90 to 1.95)
Unemployed	85.4	11.44 (6.48 to 20.21)	6.1	0.42 (0.20 to 0.89)
*Working Hours (per week)*				
0–40	54.6	Ref.	9.5	Ref.
41–59	37.3	0.49 (0.39 to 0.62)	12.8	1.39 (0.99 to 1.97)
≥ 60	26.7	0.30 (0.22 to 0.41)	15.8	1.79 (1.23 to 2.62)
*Annual Income (10,000 JPY)*				
0–99	67.6	Ref.	7.2	Ref.
100–299	58.2	0.67 (0.45 to 1.00)	9.7	1.39 (0.73 to 2.64)
300–499	40.2	0.32 (0.23 to 0.45)	10.9	1.58 (0.92 to 2.72)
500–799	29.4	0.20 (0.14 to 0.28)	14.8	2.24 (1.32 to 3.81)
800-more	13.0	0.07 (0.04 to 0.12)	19.6	3.15 (1.72 to 5.79)
*Region of Residence*				
Kanto	57.3	Ref.	6.8	Ref.
Hokkaido	50.9	1.96 (1.14 to 3.38)	8.6	0.58 (0.21 to 1.62)
Tohoku	40.6	1.51 (0.99 to 2.32)	11.1	0.75 (0.38 to 1.49)
Chubu	37.1	0.86 (0.67 to 1.11)	13.2	1.21 (0.85 to 1.73)
Kinki	36.9	0.86 (0.64 to 1.15)	11.6	1.05 (0.69 to 1.59)
Chugoku/Shikoku	38.0	0.90 (0.64 to 1.27)	16.9	1.63 (1.04 to 2.56)
Kyushu/Okinawa	39.5	0.96 (0.68 to 1.34)	16.7	1.60 (1.02 to 2.50)
*Population Density of Residence*				
Non-densely inhabited district	38.8	Ref.	16.4	Ref.
< 200,000	42.7	1.18 (0.90 to 1.53)	11.1	0.63 (0.44 to 0.92)
200,000 to < 1,000,000	37.5	0.95 (0.73 to 1.22)	12.2	0.71 (0.51 to 0.98)
≥ 1,000,000	41.6	1.12 (0.85 to 1.48)	9.1	0.51 (0.34 to 0.77)

**Table 2:** Association of sociodemographic factors with childlessness (having no children) and having 3 or more children among women born 1971–1975.

	Women (n = 2416)			
	Childlessness		3 or more children	
	Row %	aOR (95% CI)	Row %	aOR (95% CI)
*Education*				
No university degree	27.4	Ref.	15.1	Ref.
University degree	28.1	1.02 (0.76 to 1.35)	13.0	0.83 (0.59 to 1.18)
*Occupation*				
Regular employee	40.0	Ref.	10.7	Ref.
Part-time or temporary worker	21.3	0.41 (0.31 to 0.53)	17.7	1.80 (1.31 to 2.47)
Business owner or member of family business	17.8	0.32 (0.18 to 0.57)	18.0	1.84 (1.04 to 3.23)
Unemployed	23.4	0.46 (0.34 to 0.62)	13.5	1.30 (0.90 to 1.89)
*Working Hours (per week)*				
0–40	23.7	Ref.	15.9	Ref.
41–59	42.5	2.37 (1.81 to 3.11)	9.4	0.55 (0.37 to 0.80)
≥ 60	59.2	4.68 (2.26 to 9.68)	8.2	0.47 (0.11 to 1.97)
*Region of Residence*				
Kanto	40.0	Ref.	8.8	Ref.
Hokkaido	21.3	1.46 (0.83 to 2.56)	14.3	0.56 (0.23 to 1.34)
Tohoku	30.9	0.61 (0.36 to 1.02)	14.5	0.99 (0.53 to 1.83)
Chubu	22.9	0.66 (0.49 to 0.89)	14.8	1.02 (0.73 to 1.43)
Kinki	27.8	0.86 (0.63 to 1.17)	13.3	0.90 (0.62 to 1.32)
Chugoku/Shikoku	28.7	0.89 (0.60 to 1.32)	15.0	1.04 (0.64 to 1.68)
Kyushu/Okinawa	25.0	0.74 (0.51 to 1.08)	20.1	1.47 (1.00 to 2.18)
*Population Density of Residence*				
Non-densely inhabited district	22.2	Ref.	21.3	Ref.
< 200,000	29.0	1.43 (1.04 to 1.96)	12.5	0.53 (0.37 to 0.75)
200,000 to < 1,000,000	28.1	1.38 (1.03 to 1.86)	12.3	0.52 (0.38 to 0.72)
≥ 1,000,000	32.6	1.69 (1.23 to 2.31)	12.0	0.50 (0.35 to 0.72)

## Discussion

### Summary of main findings

In this analysis of nationally representative survey data including men and women born 1943–1975, we found that the decline in the total fertility rate in Japan could be attributed to both an increasing proportion of the population who were childless and a lower number of children among those who had children. Men with higher income had more children across birth cohorts. While a decline in fertility had occurred in all income groups, it was more pronounced among men with low income. Similarly, men with a university degree were more likely to have children as compared to those without such a degree in most birth cohorts. Conversely, for women born in 1956–1970, those with a university degree vs those without were less likely to have children and had fewer children; this difference was no longer seen in the youngest birth cohort (those born 1971–1975).

### Findings in relation to previous studies

While studies on the fertility of men are few, low education and income has been strongly associated with childlessness among men, with this pattern being consistently observed across populations and birth cohorts [16, 18, 22]. A strong association of lower income and education with a higher likelihood of being childless was seen also among men in our study. Importantly, while the number of children in our study decreased across income groups, the increase in childlessness and decline in total fertility was more pronounced among men with lower income than those with higher income, indicating that the income related disparities in fertility outcomes has increased in more recent birth cohorts. Moreover, men born in 1971–1975 and who were unemployed or had part-time or temporary employment were more likely to be childless as compared to those with regular employment. In previous studies, lower income and precarious employment among men have been strongly associated with a higher likelihood of having no heterosexual experience [21], being single [5] and being unmarried [24]. Given that heterosexual relationships are the most common type of childbearing union and over 95% of the children are born to married couples in Japan, the association between lower income and lower fertility in our study may not be surprising [25]. It is possible that outcomes in the labor market, marriage market and fertility outcomes have common determinants such as personality, values, and health status, and the associations may not represent causality. Nonetheless, the association could potentially also be partly explained by the strong appeal of male socioeconomic status in the Japanese marriage market [26] in combination with the decline in stable employment opportunities over the past decades. In addition, high costs of childrearing may deter some couples with lower income from having their preferred number of children [6–8]. In accordance with this hypothesis, the proportion of men born in 1971–1975 who had 3 or more children increased in a stepwise fashion with increasing income.

Several studies in Europe and the US have assessed fertility by the level of education among women [13, 14, 16–19]. This area of research has received much attention, as the increasing number of women with a higher education may have implications for marriage market dynamics [27] and choices regarding family formation [6, 19]. In most European countries and the US, women with lower education tend to be the least likely to be childless at age 40 and have the highest number of children, with fertility decreasing with higher educational level. However, the educational gap in fertility has decreased during the past decades and in the Scandinavian countries, the lowest educated women are now more likely to be childless at age 40 than those with more education [16]. As such, in the Scandinavian countries, low education is associated with lower fertility not only among men but also among women, indicating that the social inequality in childbearing, which has long been observed for men, now also applies to women. It has been hypothesized that higher earning potential (as indicated by higher education) is needed to sustain a family and that the difficulties in reconciling a career with family formation for women has been overcome in these countries [16]. Moreover, studies indicate that mate preferences of men and women are converging and that economic prospects and education are increasingly important partner search criteria also for men [26, 28]. In our study, Japanese women born between 1956 and 1970 who had a university degree were consistently more likely to be childless and had fewer children than those without a university degree. However, in the most recent birth cohort assessed (those born 1971–1975), women with a university degree had similar levels of childlessness and number of children as those without. Future studies are needed to assess whether this finding persists in younger birth cohorts and whether a trend towards a reversal of the educational gap in female fertility will also be observed in Japan.

In the analysis of the youngest birth cohort (1971–1975), we found that women who lived in larger cities were the least likely to have children and to have 3 or more children. These findings are in line with studies from other parts of the world and could possibly be explained by both selective migration (individuals who have less children are more likely to move to urban areas) and contextual factors (the effect of the immediate living environment) [29].

### Limitations

Our study has a few limitations. First, as data on fertility outcomes and socioeconomic variables were self-reported, findings may have been affected by social desirability bias [30]; the risk of such a bias, however, may have been mitigated by the survey’s use of self-administered questionnaires [31]. Moreover, the estimates for total fertility in our study was similar to those calculated using nationwide vital statistics data [32]; this indicates that the validity of the fertility outcome data used in our study is high. Second, although the response rate in the National Fertility Survey was high (70.0–77.7% among unmarried individuals and 85.7–91.1% among married couples) and the sample was weighted so that it was representative of the Japanese population in terms of sex, age, and marital status, non-response might have introduced bias in our results. Third, data on income before study participants had children were not available. As indicated by the association of having regular employment with a lower likelihood of having children among women born 1971–1975, many women with regular employment before childbirth start working part-time or become homemakers after they have children [9]. Therefore, we did not assess the association of income with fertility outcomes among women. Finally, in accordance with previous studies, we assessed fertility outcomes at age 40–44 years or 45–49 years. As some individuals, in particular men, may go on to have more children later in life, our analyses may not have included all children to individuals in the investigated birth cohorts. In our study, fertility outcome data were available at age 40–44 and 45–49 for two of the birth cohorts (1961–1965 and 1966–1970): for women, fertility outcomes were largely similar at both age ranges while for men born 1961–1965, childlessness was less common (30.6% vs 35.3%) in the older age range. The age of having children has increased during the past decades in Japan and an increasing proportion of the children born in Japan have parents in their 40s [33]; future studies are needed to assess fertility outcomes throughout the life course.

### Conclusions

We found that lower education and income were associated with a higher likelihood of being childless and having fewer children among men in Japan and that the disparity in fertility by income had increased in more recent birth cohorts. Among women, higher education was associated with lower fertility, although this pattern was no longer observed among those born in 1971–1975.

## Supporting information

S1 File Additional methods and data. (DOCX)
