# Supplementary material for: Salaries, degrees, and babies: Trends in fertility by income and education among Japanese men and women born 1943–1975—Analysis of national surveys
Source: PLoS One. 2022 Apr 27;17(4):e0266835. doi: 10.1371/journal.pone.0266835 (PMC9045600; doi:10.1371/journal.pone.0266835)
Supplement: S1 File — (DOCX) [file pone.0266835.s001.docx]

Ghaznavi et al. Salaries, degrees, and babies: trends in fertility by income and education among Japanese men and women born 1943-1975 - analysis of national surveys

**S1 File: Online Supplementary Material**

**Questions used in the National Fertility Survey**

*Survey for married couples*

| Survey year | Question in the questionnaire | Wording |
| --- | --- | --- |
| 1987 | C043 | Total number of children born |
| 1992 | C061 | Total number of children born |
| 2005 | Q10A1 | Total number of live births |
| 2010 | Q11A1 | Total number of live births |
| 2015 | Q9A1 | Total number of live births |

Married respondents were asked both about their current number of living children and the number of children to which they had given birth. For those with missing data on the number of children to which they had given birth, we used data on the number of living children. Unmarried individuals who were never married and had missing data on their number of children were assumed to have 0 children.

*Survey for unmarried individuals*

| Survey | Question in the questionnaire | Wording |
| --- | --- | --- |
| 1987 | S025 | Number of children from previous marriage |
| 1992 | S038 | Number of children born |
| 2005 | Q8C | Number of children born from first marriage |
| 2010 | Q10A1 | Number of live births so far |
| 2015 | Q15A1 | Number of live births so far |

**Calculation of sample weights**

The methods for calculation of sample weights is described in detail elsewhere.^1,2^ In brief, we used data from the Population Census to obtain information about the number of individuals by sex, age and marital status (married vs. not married) for each survey year, and calculated sample weights so that each survey was representative of the Japanese population with respect to sex, age and marital status. The sample weights were calculated after exclusion of survey participants with missing data on the number of children. The sample weights were not age-standardized.

**Use of birth cohort as a continuous variable**

| Birth cohort | Value used in analyses using birth cohort as a continuous variable |
| --- | --- |
| 1943-1947 | 1 |
| 1948-1952 | 2 |
| 1956-1960 | 3.6 |
| 1961-1965 | 4.6 |
| 1966-1970 | 5.6 |
| 1971-1975 | 6.6 |

When we used birth cohort as a continuous variable in the analyses of trends in fertility outcomes across birth cohorts, we accounted for the irregular interval between the 5-year birth cohorts used in our study (STable 1). This was done by using a continuous variable that corresponded to the calendar time interval between the birth cohorts as described in the table below.

**STable 1** Age by birth year at each survey.

|  |  | Survey year | | | |
| --- | --- | --- | --- | --- | --- |
| Birth cohort | Birth year | 1992 | 2005 | 2010 | 2015 |
| 1943-1947 | 1943 | 49 | - | - | - |
|  | 1944 | 48 | - | - | - |
|  | 1945 | 47 | - | - | - |
|  | 1946 | 46 | - | - | - |
|  | 1947 | 45 | - | - | - |
| 1948-1952 | 1948 | 44 | - | - | - |
|  | 1949 | 43 | - | - | - |
|  | 1950 | 42 | - | - | - |
|  | 1951 | 41 | - | - | - |
|  | 1952 | 40 | - | - | - |
| Not included in the analyses | 1953 | - | - | - | - |
|  | 1954 | - | - | - | - |
|  | 1955 | - | - | - | - |
| 1956-1960 | 1956 | - | 49 | - | - |
|  | 1957 | - | 48 | - | - |
|  | 1958 | - | 47 | - | - |
|  | 1959 | - | 46 | - | - |
|  | 1960 | - | 45 | - | - |
| 1961-1965 | 1961 | - | 44 | 49 | - |
|  | 1962 | - | 43 | 48 | - |
|  | 1963 | - | 42 | 47 | - |
|  | 1964 | - | 41 | 46 | - |
|  | 1965 | - | 40 | 45 | - |
| 1966-1970 | 1966 | - | - | 44 | 49 |
|  | 1967 | - | - | 43 | 48 |
|  | 1968 | - | - | 42 | 47 |
|  | 1969 | - | - | 41 | 46 |
|  | 1970 | - | - | 40 | 45 |
| 1971-1975 | 1971 | - | - | - | 44 |
|  | 1972 | - | - | - | 43 |
|  | 1973 | - | - | - | 42 |
|  | 1974 | - | - | - | 41 |
|  | 1975 | - | - | - | 40 |

**STable** **2** Number of men and women who were included in and excluded from the study population in the analyses from each round of the National Fertility Survey.

|  | *Men* | | *Women* | |
| --- | --- | --- | --- | --- |
|  | *Included* | *Excluded* | *Included* | *Excluded* |
| *Survey year* | *n (n unmarried)* | *n (n unmarried)* | *n (n unmarried)* | *N (n unmarried)* |
| 1992 | 4811 (453) | 82 (30) | 4902 (442) | 103 (32) |
| 2005 | 3256 (471) | 219 (185) | 3612 (520) | 93 (39) |
| 2010 | 4481 (1018) | 57 (21) | 4592 (842) | 78 (18) |
| 2015 | 4180 (1000) | 97 (59) | 4522 (928) | 99 (55) |

**STable 3** Number of study participants by birth cohort and survey.

|  | *Women* | | *Men* | |
| --- | --- | --- | --- | --- |
|  | *Age range* | | *Age range* | |
| *Birth cohort* | *40-44* | *45-49* | *40-44* | *45-49* |
| 1943-1947 | - | 2155 | - | 2161 |
| 1948-1952 | 2656 | - | 2741 | - |
| 1956-1960 | - | 1562 | - | 1742 |
| 1961-1965 | 1694 | 2144 | 1870 | 2270 |
| 1966-1970 | 2337 | 1948 | 2322 | 2106 |
| 1971-1975 | 2232 | - | 2416 | - |

In the primary analysis, fertility outcomes were assessed at age 45-49 years in the birth cohorts 1943-1947, 1956-1960, 1961-1965 and 1966-1970 and at age 40-44 years in the birth cohorts 1948-1952 and 1971-1975.

**STable 4** Definitions and categorization of socioeconomic and regional variables used for describing population characteristics of participants in the National Fertility Survey 2015.

| *Variable* | *Categorization* | *Missing, weighted %* |
| --- | --- | --- |
| Education | No university education; university education. | 0.66 |
| Occupational status | Regular employee; part-time or temporary worker; business owner or member of family business; unemployed (including students). | 4.00 |
| Working hours (per week)^a^ | 0-40; 41-59; ≥60 | 2.74 |
| Annual income in 10,000 Japanese Yen (JPY)^b^ | 0-99; 100-299; 300-499; 500-799; ≥800 | 5.03 (men only) |
| Region of residence^d^ | Hokkaido; Tohoku; Kanto; Chubu; Kinki; Chugoku/Shikoku; Kyushu/Okinawa | 0 |
| Population density and size of residence | Non-densely inhabited district; district with less than 200,000 inhabitants; between 200,000 and 1,000,000 inhabitants; more than 1,000,000 inhabitants^3^ | 0 |

**STable 5** Trends in total fertility and the distribution of children across birth cohorts in the primary analysis and at age 40-44 years and 45-49 years for men. Numbers within brackets are 95% CI.

|  |  | **Birth cohort** | | | | | |  |  |
| --- | --- | --- | --- | --- | --- | --- | --- | --- | --- |
|  | *Number of children* | *1943-1947* | *1948-1952* | *1956-1960* | *1961-1965* | *1966-1970* | *1971-1975* | *aOR (95% CI) for trend* | *Coefficient (95% CI) for trend in total fertility* |
| **Primary analysis*** | 0, % | 14.3  (12.3 to 16.6) | 19.5 (17.8 to 21.3) | 29.9  (27.2 to 32.7) | 30.6  (28.4 to 32.8) | 34.7  (32.4 to 37.0) | 39.9 (37.8 to 42.2) | 1.26 (1.23 to 1.30) | - |
|  | 1, % | 9.1 (7.8 to 10.6) | 10.0 (8.9 to 11.3) | 11.3 (9.9 to 12.9) | 15.1 (13.6 to 16.7) | 16.8 (15.2 to 18.6) | 17.1 (15.6 to 18.7) | 1.15 (1.12 to 1.19) | - |
|  | 2, % | 52.5 (49.9 to 55.1) | 44.1 (42.2 to 46.1) | 37.5 (35.1 to 40.0) | 38.5 (36.4 to 40.6) | 35.6 (33.4 to 37.8) | 30.5 (28.6 to 32.5) | 0.86 (0.85 to 0.88) | - |
|  | 3 or more, % | 24.1 (22.0 to 26.3) | 26.3 (24.7 to 28.1) | 21.3 (19.4 to 23.4) | 15.8 (14.3 to 17.4) | 12.9 (11.5 to 14.5) | 12.5 (11.2 to 13.9) | 0.84 (0.82 to 0.87) | - |
|  | Total fertility | 1.92 (1.86 to 1.98) | 1.82 (1.77 to 1.86) | 1.53 (1.46 to 1.59) | 1.42 (1.37 to 1.47) | 1.29  (1.24 to 1.35) | 1.17 (1.13 to 1.22) | - | -0.14 (-0.15 to -0.13) |
| **40-44 years** | 0, % | - | 19.5 (17.8 to 21.3) | - | 35.3 (32.8 to 37.9) | 37.0 (34.9 to 39.0) | 39.9 (37.8 to 42.1) | 1.25 (1.21 to 1.29) | - |
|  | 1, % | - | 10.0 (8.9 to 11.2) | - | 12.3 (10.9 to 13.9) | 15.6 (14.2 to 17.1) | 17.1 (15.6 to 18.7) | 1.15 (1.10 to 1.19) | - |
|  | 2, % | - | 44.1 (42.2 to 46.1) | - | 36.0 (33.7 to 38.3) | 34.5 (32.6 to 36.4) | 30.5 (28.7 to 32.4) | 0.88 (0.86 to 0.91) | - |
|  | 3 or more, % | - | 26.3 (24.7 to 28.0) | - | 16.4 (14.7 to 18.2) | 13.0 (11.7 to 14.4) | 12.5 (11.2 to 13.9) | 0.81 (0.78 to 0.83) | - |
|  | Total fertility | - | 1.82 (1.77 to 1.86) | - | 1.36 (1.30 to 1.42) | 1.25 (1.21 to 1.30) | 1.17 (1.13 to 1.22) | - | -0.14 (-0.16 to -0.13) |
| **45-49 years** | 0, % | 14.3 (12.3 to 16.6) | - | 29.9 (27.1 to 32.7) | 30.6 (28.4 to 32.8) | 34.7 (32.4 to 37.0) | - | 1.28 (1.23 to 1.34) | - |
|  | 1, % | 9.1 (7.8 to 10.6) | - | 11.3 (9.9 to 12.9) | 15.1 (13.6 to 16.7) | 16.8 (15.2 to 18.5) | - | 1.17 (1.11 to 1.23) | - |
|  | 2, % | 52.5 (49.9 to 55.1) | - | 37.5 (35.1 to 40.0) | 38.5 (36.4 to 40.6) | 35.6 (33.5 to 37.8) | - | 0.86 (0.83 to 0.88) | - |
|  | 3 or more, % | 24.1 (22.0 to 26.3) | - | 21.3 (19.4 to 23.4) | 15.8 (14.3 to 17.4) | 12.9 (11.5 to 14.5) | - | 0.86 (0.83 to 0.89) | - |
|  | Total fertility | 1.92 (1.86 to 1.98) | - | 1.53 (1.46 to 1.59) | 1.42 (1.37 to 1.47) | 1.29 (1.24 to 1.35) | - | - | -0.14 (-0.16 to -0.12) |

* In the primary analysis, fertility outcomes were assessed at age 45-49 years in the birth cohorts 1943-1947, 1956-1960, 1961-1965 and 1966-1970 and at age 40-44 years in the birth cohorts 1948-1952 and 1971-1975
aOR = age-adjusted odds ratio

**STable 6** Trends in total fertility and the distribution of children across birth cohorts in the primary analysis and at age 40-44 years and 45-49 years for women. Numbers within brackets are 95% CI.

|  |  | **Birth cohort** | | | | | |  |  |
| --- | --- | --- | --- | --- | --- | --- | --- | --- | --- |
|  | *Number of children* | *1943-1947* | *1948-1952* | *1956-1960* | *1961-1965* | *1966-1970* | *1971-1975* | *aOR (95% CI) for trend* | *Coefficient (95% CI) for trend in total fertility* |
| **Primary analysis*** | 0, % | 11.6 (9.8 to 13.6) | 12.2  (10.9 to 13.7) | 16.4  (14.7 to 18.4) | 21.0  (19.2 to 22.9) | 25.9  (24.0 to 28.0) | 27.6  (25.6 to 29.6) | 1.23 (1.20 to 1.27) | - |
|  | 1, % | 10.6  (9.1 to 12.3) | 10.7  (9.5 to 12.0) | 13.0  (11.5 to 14.8) | 14.6  (13.1 to 16.1) | 17.8  (16.1 to 19.5) | 20.3  (18.6 to 22.0) | 1.16 (1.12 to 1.19) | - |
|  | 2, % | 52.9 (50.3 to 55.5) | 50.6  (48.6 to 52.6) | 42.5  (40.2 to 44.9) | 43.0  (40.9 to 45.1) | 39.5  (37.4 to 41.7) | 37.4  (35.4 to 39.4) | 0.89 (0.87 to 0.91) | - |
|  | 3 or more, % | 24.9  (22.8 to 27.2) | 26.5  (24.8 to 28.2) | 28.0  (25.9 to 30.2) | 21.5  (19.8 to 23.3) | 16.8  (15.2 to 18.5) | 14.7  (13.4 to 16.3) | 0.89 (0.87 to 0.91) | - |
|  | Total fertility | 1.96  (1.91 to 2.01) | 1.96  (1.92 to 2.00) | 1.86  (1.81 to 1.92) | 1.68  (1.64 to 1.73) | 1.50  (1.45 to 1.55) | 1.42  (1.37 to 1.46) | - | -0.10 (-0.12 to -0.09) |
| **40-44 years** | 0, % | - | 12.2  (10.9 to 13.7) | - | 22.2  (20.2 to 24.4) | 26.4  (24.5 to 28.4) | 27.6  (25.7 to 29.6) | 1.25 (1.21 to 1.29) | - |
|  | 1, % | - | 10.7  (9.5 to 12.0) | - | 15.0  (13.5 to 16.8) | 18.4  (16.9 to 20.1) | 20.3  (18.7 to 21.9) | 1.18 (1.14 to 1.23) | - |
|  | 2, % | - | 50.6  (48.7 to 52.6) | - | 42.1  (39.9 to 44.5) | 40.0  (38.0 to 42.1) | 37.4  (35.5 to 39.4) | 0.89 (0.87 to 0.91) | - |
|  | 3 or more, % | - | 26.5  (24.8 to 28.2) | - | 20.6  (18.8 to 22.5) | 15.1  (13.7 to 16.6) | 14.7  (13.4 to 16.2) | 0.85 (0.82 to 0.87) | - |
|  | Total fertility | - | 1.96  (1.92 to 2.00) | - | 1.65  (1.60 to 1.70) | 1.46  (1.42 to 1.51) | 1.42  (1.37 to 1.46) | - | -0.12 (-0.14 to -0.11) |
| **45-49 years** | 0, % | 11.6  (9.9 to 13.6) | - | 16.4  (14.6 to 18.4) | 21.0  (19.2 to 22.9) | 25.9  (24.0 to 28.0) | - | 1.24 (1.18 to 1.30) | - |
|  | 1, % | 10.6  (9.2 to 12.2) | - | 13.0  (11.5 to 14.8) | 14.6  (13.1 to 16.1) | 17.8  (16.2 to 19.5) | - | 1.13 (1.08 to 1.19) | - |
|  | 2, % | 52.9  (50.3 to 55.4) | - | 42.5  (40.2 to 44.9) | 43.0  (40.9 to 45.1) | 39.5  (37.4 to 41.7) | - | 0.89 (0.86 to 0.92) | - |
|  | 3 or more, % | 24.9  (22.8 to 27.2) | - | 28.0  (25.9 to 30.2) | 21.5  (19.9 to 23.3) | 16.8  (15.2 to 18.5) | - | 0.92 (0.89 to 0.95) | - |
|  | Total fertility | 1.96  (1.91 to 2.01) | - | 1.86  (1.81 to 1.92) | 1.68  (1.63 to 1.73) | 1.50  (1.45 to 1.55) | - | - | -0.09 (-0.11 to -0.08) |

* In the primary analysis, fertility outcomes were assessed at age 45-49 years in the birth cohorts 1943-1947, 1956-1960, 1961-1965 and 1966-1970 and at age 40-44 years in the birth cohorts 1948-1952 and 1971-1975
aOR = age-adjusted odds ratio

**STable 7** Number of children and total fertility among men and women by education and birth cohort.

|  | **No university education** | | | | | **University education** | | | | | **Comparison by education** | |
| --- | --- | --- | --- | --- | --- | --- | --- | --- | --- | --- | --- | --- |
|  | Number of children, % | | | |  | Number of children, % | | | |  |  |  |
| **Birth cohort** | 0 | 1 | 2 | 3 or more | Total fertility | 0 | 1 | 2 | 3 or more | Total fertility | p-value for difference in childlessness | p-value for difference in total fertility |
| *Men* |  |  |  |  |  |  |  |  |  |  |  |  |
| 1943-1947 | 15.0 | 8.7 | 52.8 | 23.5 | 1.91 | 11.9 | 9.9 | 52.1 | 26.1 | 1.99 | 0.426 | 0.226 |
| 1948-1952 | 20.9 | 9.2 | 43.5 | 26.4 | 1.80 | 15.7 | 12.3 | 45.6 | 26.4 | 1.85 | 0.009 | 0.339 |
| 1956-1960 | 34.7 | 9.5 | 33.5 | 22.4 | 1.46 | 22.9 | 13.8 | 43.7 | 19.6 | 1.62 | <0.001 | 0.035 |
| 1961-1965 | 32.2 | 13.0 | 37.3 | 17.6 | 1.43 | 27.4 | 18.4 | 41.2 | 13.0 | 1.42 | 0.001 | 0.798 |
| 1966-1970 | 36.8 | 15.0 | 33.9 | 14.3 | 1.29 | 30.4 | 20.2 | 38.9 | 10.5 | 1.31 | 0.001 | 0.692 |
| 1971-1975 | 42.2 | 15.6 | 29.2 | 13 | 1.15 | 35.5 | 20.0 | 32.9 | 11.6 | 1.22 | 0.007 | 0.194 |
| *Women* |  |  |  |  |  |  |  |  |  |  |  |  |
| 1943-1947 | 11.2 | 10.5 | 53.5 | 24.8 | 1.97 | 15.7 | 12.7 | 46.9 | 24.7 | 1.85 | 0.546 | 0.368 |
| 1948-1952 | 12.0 | 10.5 | 51.0 | 26.5 | 1.96 | 15.2 | 12.8 | 47.6 | 24.5 | 1.84 | 0.456 | 0.167 |
| 1956-1960 | 15.9 | 12.2 | 42.5 | 29.4 | 1.90 | 19.5 | 18.0 | 43.5 | 19.1 | 1.65 | 0.008 | 0.004 |
| 1961-1965 | 19.6 | 14.4 | 43.3 | 22.7 | 1.73 | 28.3 | 16.1 | 40.7 | 14.9 | 1.46 | 0.002 | 0.001 |
| 1966-1970 | 25.0 | 16.9 | 40.0 | 18.1 | 1.54 | 31.3 | 21.9 | 36.7 | 10.1 | 1.27 | 0.002 | <0.001 |
| 1971-1975 | 27.4 | 20.3 | 37.1 | 15.1 | 1.42 | 28.1 | 19.8 | 39.0 | 13.0 | 1.39 | 0.779 | 0.605 |

**STable 8** Number of children and total fertility among men by annual income category and birth cohort.

|  |  | Number of children, % | | | |  |  |  |
| --- | --- | --- | --- | --- | --- | --- | --- | --- |
| Annual income (10,000 JPY) | Birth cohort | 0 | 1 | 2 | 3 or more | Total fertility | p-value for difference in childlessness across income groups | p-value for difference in total fertility vs income category 0 to <300 |
| 0 to <300 | 1943-1947 | 25.7 | 9.0 | 40.7 | 24.5 | 1.74 | < 0.001 | Ref |
|  | 1948-1952 | 30.3 | 11.6 | 35.3 | 22.9 | 1.55 | < 0.001 | Ref |
|  | 1956-1960 | 55.0 | 7.5 | 24.5 | 13.0 | 0.97 | < 0.001 | Ref |
|  | 1961-1965 | 52.2 | 12.5 | 22.9 | 12.4 | 0.98 | < 0.001 | Ref |
|  | 1966-1970 | 57.5 | 14.3 | 19.6 | 8.6 | 0.81 | < 0.001 | Ref |
|  | 1971-1975 | 62.8 | 12.1 | 16.6 | 8.5 | 0.73 | < 0.001 | Ref |
| 300 to <600 | 1943-1947 | 18.3 | 9.0 | 51.5 | 21.2 | 1.79 | < 0.001 | 0.686 |
|  | 1948-1952 | 20.8 | 9.2 | 43.5 | 26.5 | 1.80 | < 0.001 | 0.002 |
|  | 1956-1960 | 27.8 | 11.4 | 37.7 | 23.1 | 1.58 | < 0.001 | < 0.001 |
|  | 1961-1965 | 27.1 | 13.8 | 41.4 | 17.6 | 1.52 | < 0.001 | < 0.001 |
|  | 1966-1970 | 34.3 | 15.6 | 36.3 | 13.8 | 1.33 | < 0.001 | < 0.001 |
|  | 1971-1975 | 38.8 | 17.6 | 32.3 | 11.2 | 1.17 | < 0.001 | < 0.001 |
| ≥600 | 1943-1947 | 6.9 | 8.5 | 59.2 | 25.3 | 2.10 | < 0.001 | 0.005 |
|  | 1948-1952 | 11.7 | 10.1 | 51.4 | 26.8 | 1.97 | < 0.001 | < 0.001 |
|  | 1956-1960 | 16.7 | 13.1 | 46.5 | 23.7 | 1.80 | < 0.001 | < 0.001 |
|  | 1961-1965 | 19.2 | 17.5 | 47.3 | 16.0 | 1.62 | < 0.001 | < 0.001 |
|  | 1966-1970 | 17.4 | 20.0 | 47.9 | 14.7 | 1.63 | < 0.001 | < 0.001 |
|  | 1971-1975 | 20.0 | 20.8 | 41.2 | 18.0 | 1.60 | < 0.001 | < 0.001 |

**STable 9** Trends in childlessness (having no children) and total fertility by education (men and women) and income group (men).

|  | **Men** | | | | **Women** | | | |
| --- | --- | --- | --- | --- | --- | --- | --- | --- |
|  | Childlessness | | Total fertility | | Childlessness | | Total fertility | |
|  | aOR (95% CI) for trend | P-value for interaction | Coefficient (95% CI) for trend | P-value for interaction | aOR (95% CI) | P-value for interaction | Coefficient (95% CI) for trend | P-value for interaction |
| *Education* |  |  |  |  |  |  |  |  |
| No university education | 1.27 (1.23 to 1.31) | Ref | -0.14 (-0.15 to -0.12) | Ref | 1.24 (1.20 to 1.28) | Ref | -0.10 (-0.11 to -0.09) | Ref |
| University education | 1.27 (1.20 to 1.35) | 0.944 | -0.14 (-0.17 to -0.12) | 0.672 | 1.18 (1.08 to 1.28) | 0.295 | -0.10 (-0.14 to -0.06) | 0.902 |
| *Annual income (10,000 JPY)* |  |  |  |  |  |  |  |  |
| 0 to <300 | 1.32 (1.23 to 1.43) | Ref | -0.18 (-0.22 to -0.14) | Ref | - | - | - | - |
| 300 to <600 | 1.20 (1.15 to 1.26) | 0.012 | -0.12 (-0.13 to -0.10) | 0.001 | - | - | - | - |
| ≥600 | 1.21 (1.14 to 1.29) | 0.044 | -0.10 (-0.12 to -0.08) | <0.001 | - | - | - | - |

**STable 10** Population characteristics by number of children among men and women born 1971-1975. Numbers are shown as column %.

|  | **Number of Children** | | | | | | | | |
| --- | --- | --- | --- | --- | --- | --- | --- | --- | --- |
|  | **Men** | | | | | **Women** | | | |
|  | **0** | **1** | **2** | **3 or more** | **0** | | **1** | **2** | **3 or more** |
| Unweighted n | 729 | 423 | 767 | 313 | 556 | | 510 | 972 | 378 |
| *Education* |  |  |  |  |  | |  |  |  |
| No university education | 69.4 | 59.8 | 62.9 | 68.1 | 83.0 | | 83.7 | 82.6 | 85.3 |
| University education | 30.6 | 40.2 | 37.1 | 31.9 | 17.0 | | 16.3 | 17.4 | 14.7 |
| *Occupation* |  |  |  |  |  | |  |  |  |
| Regular employee | 63.8 | 82.0 | 83.1 | 78.7 | 44.6 | | 29.3 | 24.0 | 22.3 |
| Part-time or temporary worker | 13.5 | 3.1 | 2.9 | 1.8 | 32.1 | | 36.9 | 46.7 | 49.9 |
| Business owner or member of family business | 10.3 | 13.6 | 13.0 | 16.8 | 3.5 | | 6.5 | 5.7 | 6.7 |
| Unemployed | 12.4 | 1.3 | 0.9 | 2.8 | 19.7 | | 27.2 | 23.7 | 21.2 |
| *Working Hours (per week)* |  | |  |  |  | |  |  |  |
| 0-40 | 43.5 | 25.5 | 24.1 | 24.8 | 69.3 | | 82.7 | 85.3 | 87.8 |
| 41-59 | 42.2 | 48.7 | 48.8 | 47.3 | 27.1 | | 16.1 | 13.9 | 11.3 |
| ≥ 60 | 14.3 | 25.8 | 27.0 | 27.8 | 3.6 | | 1.2 | 0.8 | 0.9 |
| *Annual Income (10,000 JPY)* |  |  |  |  |  | |  |  |  |
| 0-99 | 21.5 | 9.6 | 5.4 | 7.6 | 0.0 | | 0.0 | 0.0 | 0.0 |
| 100-299 | 19.7 | 9.4 | 9.2 | 10.9 | 0.0 | | 0.0 | 0.0 | 0.0 |
| 300 -499 | 33.7 | 34.8 | 35.1 | 30.2 | 0.0 | | 0.0 | 0.0 | 0.0 |
| 500-799 | 22.3 | 35.1 | 37.0 | 37.2 | 0.0 | | 0.0 | 0.0 | 0.0 |
| 800 or more | 2.8 | 11.0 | 13.4 | 14.1 | 0.0 | | 0.0 | 0.0 | 0.0 |
| *Region of Residence* |  |  |  |  |  | |  |  |  |
| Hokkaido | 4.1 | 3.0 | 1.7 | 1.5 | 4.5 | | 3.6 | 2.3 | 1.8 |
| Tohoku | 7.4 | 3.4 | 5.8 | 4.0 | 4.7 | | 7.1 | 6.6 | 5.9 |
| Kanto | 34.3 | 35.7 | 33.4 | 30.1 | 37.3 | | 32.1 | 31.2 | 32.7 |
| Chubu | 20.8 | 22.2 | 24.1 | 23.6 | 17.6 | | 20.7 | 24.1 | 21.2 |
| Kinki | 14.8 | 16.9 | 17.5 | 14.9 | 17.9 | | 19.1 | 17.7 | 16.1 |
| Chugoku/Shikoku | 9.0 | 9.2 | 8.8 | 12.8 | 9.3 | | 9.1 | 8.5 | 9.1 |
| Kyushu/Okinawa | 9.7 | 9.6 | 8.7 | 13.1 | 8.7 | | 8.3 | 9.7 | 13.1 |
| *Population Density of Residence* |  |  |  |  |  | |  |  |  |
| Non-densely inhabited district | 27.4 | 26.8 | 26.4 | 37.0 | 21.7 | | 24.3 | 27.7 | 39.1 |
| < 200,000 | 26.3 | 17.7 | 27.4 | 21.9 | 24.5 | | 23.4 | 23.9 | 19.9 |
| 200,000 to < 1,000,000 | 25.8 | 30.5 | 28.3 | 26.8 | 30.0 | | 30.2 | 30.7 | 24.7 |
| ≥ 1,000,000 | 20.5 | 25.0 | 17.9 | 14.3 | 23.7 | | 22.2 | 17.8 | 16.3 |

**SFigure 1** Distribution of annual income for men (in 10 000 JPY) and education for men and women by birth cohort.

*
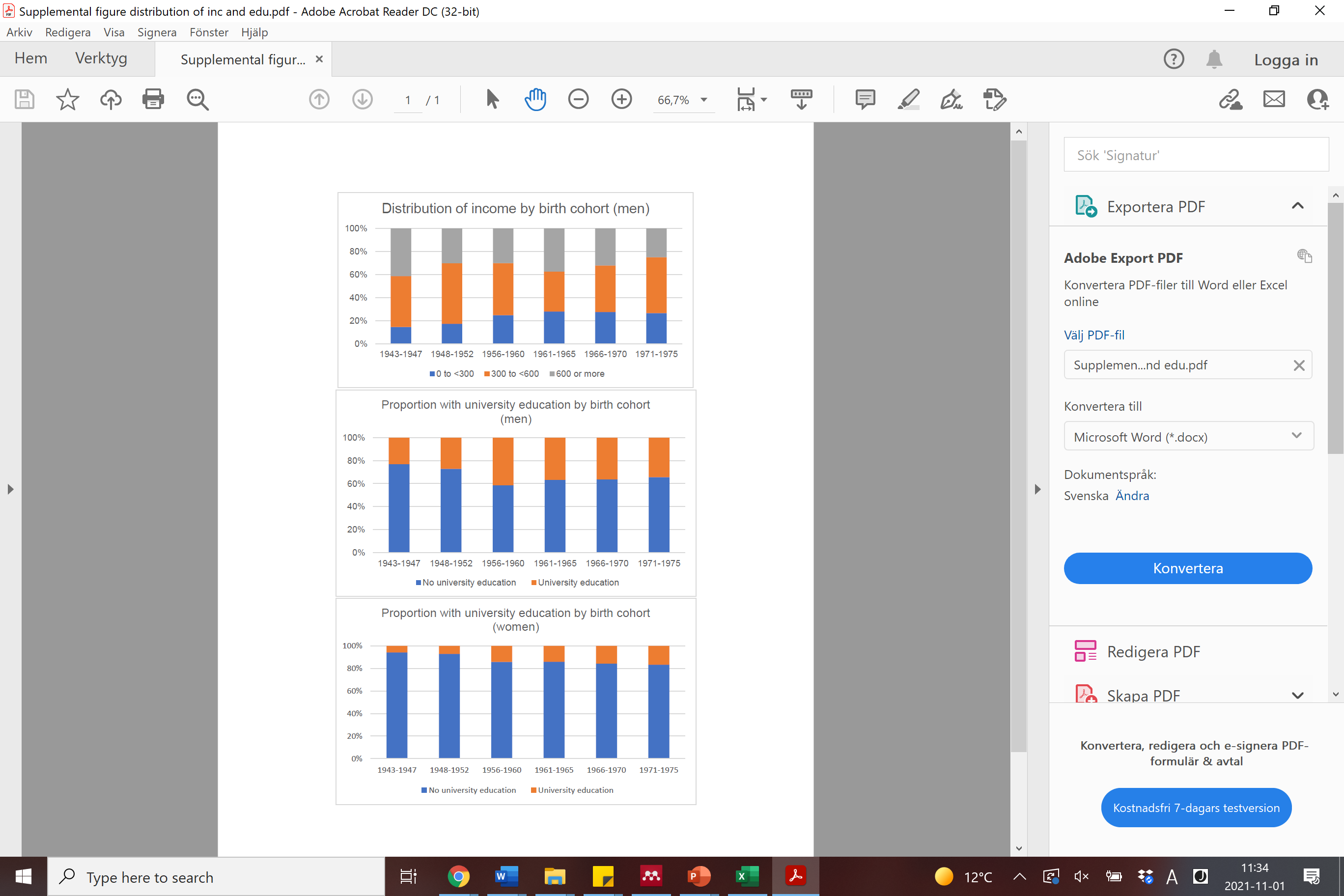
*

**References**

1. Ghaznavi C, Sakamoto H, Yoneoka D, Nomura S, Shibuya K, Ueda P. Trends in heterosexual inexperience among young adults in Japan: analysis of national surveys, 1987–2015. *BMC Public Health*. 2019;19(1):355.

2. Ghaznavi C, Sakamoto H, Nomura S, et al. The herbivore’s dilemma: Trends in and factors associated with heterosexual relationship status and interest in romantic relationships among young adults in Japan—Analysis of national surveys, 1987–2015. Panzeri M, ed. *PLoS One*. 2020;15(11):e0241571.

3. Statistics Japan. Statistics Bureau, Ministry of Internal Affairs and Communications. What is a Densely Inhabited District?
